# The Effects of Slaughter Methods and Drying Temperatures on the Protein Hydrolysis of Black Soldier Fly Larvae Meal

**DOI:** 10.3390/ani14111709

**Published:** 2024-06-06

**Authors:** María Rodríguez-Rodríguez, María José Sánchez-Muros, María del Carmen Vargas-García, Ágnes Timea Varga, Dmitri Fabrikov, Fernando García Barroso

**Affiliations:** 1 Department of Biology and Geology, CECOUAL, University of Almería, Carretera de Sacramento s/n, 04120 Almería, Spain; mrr960@ual.es (M.R.-R.); fbarroso@ual.es (F.G.B.); 2 Department of Biology and Geology, CEIMAR, University of Almería, Carretera de Sacramento s/n, 04120 Almería, Spain; mjmuros@ual.es (M.J.S.-M.); df091@ual.es (D.F.)

**Keywords:** insect processing, insect meal, digestibility, hydrolysis degree, *Hermetia illucens*

## Abstract

**Simple Summary:**

Insects, because of their protein content and environmental advantages, have been considered a promising alternative to other traditional protein sources. The black soldier fly (BSFL) is one of the most studied insects given its use as an alternative feed for farmed animals (mammals, poultry, and fish). This study investigated the effect of the method of slaughter (freezing, blanching, Melacide^®^ + freezing, and liquid nitrogen slaughter) and drying temperature (50, 70, and 90 °C) on the protein digestibility, proximal composition, and organic matter digestibility of BSFL meal. The results show that the best slaughter methods were slaughtering with liquid nitrogen and blanching, and 70 °C was the drying temperature that showed the best digestibility results while maintaining good hygienic–sanitary conditions.

**Abstract:**

In recent years, the potential of insects as a sustainable protein alternative to feed the growing world population has been explored. Differences in the ways insects are processed can affect their proximate composition and digestibility. This work studied the effects of the combination of different types of slaughter methods and drying temperatures on the proximate composition, organic matter digestibility (OMd), hydrolysis degree (DH/NH_2_ and DH/100 g DM), total hydrolysis (TH), and hygienic and sanitary characteristics of BSFL (black soldier fly larvae) meal. Four types of slaughter methods were used including freezing (F), blanching + freezing (B), Melacide^®^ + freezing (M), and liquid nitrogen slaughter (N). Each of these was used with three drying temperatures (50, 70, and 90 °C). A negative correlation between the acid detergent fiber (ADF) and protein digestibility parameters was obtained. The most suitable drying temperature was 70 °C, as it produced higher values of protein digestibility (DH and TH), resulting in hygienic and sanitary conditions suitable for food use. Slaughtering with liquid nitrogen and blanching was more conducive to achieving high protein digestibility results than traditional freezing or the use of Melacide^®^.

## 1. Introduction

The increase in the world population in recent years has resulted in a search for more sustainable protein sources to cope with the increase in protein demand [1]. Insects have been reported to be a promising alternative because of their potential to be fed with organic waste, low water consumption, high efficiency in feed conversion, and low emissions of greenhouse gases and ammonia, as well as posing few animal welfare problems and a low risk of transmission of zoonotic infections [2]. In addition, they have a high protein content and a good amino acid composition [3].

One of the most studied and mass-produced species is *Hermetia illucens* (Linneo, 1758) (black soldier fly larvae) [4]. Both industry professionals and researchers have shown interest in this species because of its potential to contribute to the circular economy through the waste management of a wide range of decomposing organic materials (food or agricultural waste), as it consumes 25–500 mg of fresh matter/larva/day, as well as being a source of protein [5,6,7]. It is a dipteran with a life cycle that includes the following stages: egg, larva, prepupa, pupa, and adult. However, the prepupal and larval stages are the most widely used in research as an alternative feed because, at these stages, the insect is a good source of protein (40–56.9% in dry matter), lipids (35–40% in DM), and minerals [8,9]. For these reasons, its inclusion in the diet of terrestrial and aquatic animals has been studied [8,9,10,11]. This is the case for farm animals, such as piglets, chickens, ducks, and quail, and farmed fish such as seabass, turbot, jian carp, rainbow trout, and shrimp [2,9,12,13]. The optimal level of inclusion of BSFL seems to vary by species [11], with full inclusion achieved for Atlantic salmon without compromising growth or sensory qualities [14].

If new protein sources are used as food and feed, their digestibility should be assessed, as protein quality is determined by amino acid composition and digestibility. In a recent review, Rodríguez-Rodríguez et al. [15] noted that BSFL showed very good results for crude protein digestibility (CPd), with a maximum of 89.7% [16] and lower for prepupae, at 50% [12], using commercial enzymes simulating dog and duck digestion, respectively. These results were obtained for raw insects; however, if the aim is to market insect meal for human consumption, it is vitally important to determine the most appropriate processing method. Kinyuru et al. [17] stated that processing methods can affect the nutritional composition of insects, especially the digestibility of their proteins.

Processing methods aim to improve the texture, palatability, nutrient bioavailability, and food safety of insect meals [15]. Although in recent years, several researchers have studied the effect of treatments, such as killing methods, drying methods, defatting, or extrusion, on the nutritional value of BSFL [18,19,20,21], results indicating the impact of different processing techniques or combinations thereof on their nutritional value or digestibility are still limited. As a result, a standard procedure for processing BSFL meal has not yet been established.

Currently, the method most companies use to slaughter different insect species is slow freezing, which can lead to the enzymatic browning of insects [20]. This browning is caused by the action of endogenous phenol oxidases (POs) that oxidize monophenols and diphenols to form o-quinones [19], which act during slow freezing. In living insects, this browning is a defense reaction or sclerotization [22]. In addition to the color change, this browning could affect both protein quality and digestibility [20]. This browning can be prevented by the use of chemical inhibitors (such as ascorbic acid, sulfite, or Melacide^®^) or physical treatments (boiling, blanching, or sonication) [23].

Another critical process is heat treatment during drying prior to grinding, as protein-rich insects can show non-enzymatic browning (Maillard reaction), which can affect the nutritional value of BSFL meal [24,25]. Depending on the temperature, heat treatment could improve protein digestibility by denaturing, increasing enzyme susceptibility, or inactivating possible inhibitors of specific enzymes [26]. However, if the temperatures are too high, reactions among amino acids can occur, which prevent hydrolysis [27].

Therefore, it is necessary to investigate the optimal processing methods for each insect species. The aim of this work is to determine the effects of different types of slaughter methods and drying temperatures on the nutritional value and protein digestibility of BSFL meals as a feed source for farm animals, in addition to the evaluation of their hygienic–sanitary quality.

## 2. Materials and Methods

BSFL were purchased from Entomo AgroIndustrial (Cehegín, Murcia, Spain). The insects were divided into lots of 375 g and subjected to different types of slaughter as follows. Freezing: without any additional treatment, live larvae were placed in zipper bags in the freezer at −18 °C. Blanching: larvae were placed in boiling water at 100 °C for 1 min and then removed from the water with a strainer and placed in zipper bags in the freezer at −18 °C. Melacide^®^ (Tequisa, Pontevedra, Spain): an antioxidant, antimelanosis, color, and texture stabilizer additive used for frozen crustaceans. This treatment was carried out precisely to try to reduce the possible melanization of the insects during slaughter. The larvae were mixed with Melacide^®^ (15 g per kilo of BSFL) and placed in zipper bags in the freezer at −18 °C. Liquid nitrogen: liquid nitrogen was poured into a container with the larvae. The larvae were placed in zipper bags in the freezer at −18 °C. Then, each lot was subdivided into three different lots of 125 g of fresh matter, each of which was dried at different temperatures (50, 70, and 90 °C) and ground (Table 1) to obtain at least 50 g of dry matter per sample.

In addition, soybean meal (SBM), albumin (A), and whey protein (W) were used as comparisons. Albumin and whey protein are pure proteins; they can serve as a control for the hydrolysis process. Soya, on the other hand, is a vegetable protein source traditionally used in animal feed.

### 2.1. Proximate Composition

The proximate composition was determined according to the Association of Official Analytical Chemists [28]. Dry matter and ash were determined via gravimetry after drying at 105 °C (AOAC #934.01) in a conventional oven and combustion at 500 °C in a muffle furnace (AOAC #942.05). Crude protein (CP) was determined using the Kjeldahl method (AOAC #954.01). The CP data of the BSFL pretreatments were calculated using the conversion factor of 4.76. This coefficient was used following the recommendation of Janssen et al. [29] to avoid the overestimation of protein in insects. For the remainder of the materials analyzed (albumin, whey protein, and soybean meal), the usual conversion coefficient of 6.25 was used. The contents of acid detergent fiber (ADF) were determined according to the Van Soest et al. [30] method using an Ankom^200^ fiber analyzer (Ankom Technology, Macedon, NY, USA). All analyses were performed in triplicate.

### 2.2. In Vitro Protein Hydrolysis

The protocol described by Huang et al. [18] was followed with some modifications. First, 100 g of BSFL meal was mixed with 4 mL NaCl (2 g/L) + HCl (7 mL/L), pH 2. The mixture was incubated at 38 °C for 15 min in a shaking water bath. The gastric phase was carried out using porcine pepsin (P7000 Sigma-Aldrich, St. Louis, MO, USA) for 240 min under constant agitation at 38 °C. After gastric digestion, the pH was adjusted to 7 with 0.2 M NaOH solution, and the intestinal phase was carried out using porcine pancreatin (P1750 Sigma-Aldrich) for 240 min under constant agitation at 38 °C.

Before adding enzymes from the gastric and intestinal phases, samples were taken to be used as blanks. In this way, the data from both phases were not affected by the amino acids present at time 0. As the intestinal phase occurs after the gastric phase, we considered that the intestinal digestibility included the sum of the gastric + intestinal phase.

The reaction of the collected samples was stopped with an equal volume of 20% trichloroacetic acid (TCA). Three replicates per sample were analyzed.

### 2.3. Organic Matter Digestibility (OMd)

After in vitro hydrolysis, the samples were centrifuged. They were washed with 80 mL of distilled water and centrifuged again. The residue was dried at 100 °C for 24 h to calculate the dry matter and then at 500 °C to obtain the residue ash. The following formula was used to calculate the digestibility of organic matter:

(1) $$\%\;OM\;Digestibility=\frac{{(DM}_{i}-{Ash}_{i})-{(DM}_{f}-{Ash}_{f})}{{(DM}_{i}-{Ash}_{i})}\times 100$$

where DM_i_ is the initial dry matter, DM_f_ is the final dry matter expressed in grams, Ash_i_ is the initial ash of the BSFL meal, and Ash_f_ is the final ash of the BSFL after hydrolysis.

### 2.4. O-Phthaldialdehyde (OPA) Method

The OPA methodology described by Church et al. [31] is based on the reaction of OPA and 2-mercaptoethanol with amino groups. It was used for the calculation of α-amino groups, using L-leucine as a standard. α-Amino groups are found in most amino acids that make up proteins. This method measures the α-amino groups released after proteolysis.

The OPA results were expressed as the % of the free amino groups in regard to the total amino groups (DH/NH_2_) and the % of the free amino groups in regard to the dry matter (DH/DM) according to the following calculation.

The degree of hydrolysis (DH/100 NH_2_) relates the number of peptide bonds broken during hydrolysis (h) to the total number of peptide bonds present in the sample (h_tot_). Its formula is as follows:

(2) $$DH/{NH}_{2}(\%)=\frac{h}{htot}\times 100$$

where h is calculated by measuring the free amino groups using OPA at the end of hydrolysis (480 min), and h_tot_ is the total amino groups in the sample [32]. These were determined by hydrolyzing 50 mg of the initial sample with 2.5 mL of 6 M HCl for 24 h at 100 °C.

DH/100 g of DM refers to the number of peptide bonds broken during protein hydrolysis (h) per 100 g of dry matter. Its formula is as follows:

(3) $$DH/DM(\%)=\frac{h_{\left(0,240,480\right)}}{{DM}_{i}}\times 100$$

where h is calculated by measuring the free amino groups with OPA at the beginning of hydrolysis (h_0_) and after the end of the gastric (h_240_) and intestinal (h_480_) phases, and DM_i_ is the organic matter introduced at the start of protein hydrolysis.

Time 0 was used as a blank for the gastric and intestinal phases, while the intestinal phase was the sum of the gastric and intestinal phases.

The reason for expressing the results in dry matter was to measure the yield of the sample, since the purpose of these meals is to be used in feed as a substitute for other proteins.

### 2.5. Total Hydrolysis (TH)

The degree of hydrolysis only measures the broken peptide bonds at the end of in vitro hydrolysis (480 min). However, the small peptides present that are susceptible to end hydrolysis are not considered. Therefore, to calculate total hydrolysis, the final hydrolysis supernatant was completely hydrolyzed with the same amount of 12 M HCl for 24 h at 100 °C and neutralized with 6 M NaOH. The formula for calculating total hydrolysis is as follows:

(4) $$Total\;hydrolysis(\%)=\frac{supernatant\;completely\;hydrolyzed}{htot}\times 100$$

### 2.6. Hygienic–Sanitary Quality Study: Microbial Contamination Indicators

To establish the level of the hygienic–sanitary safety provided by each of the slaughter and drying temperature combinations tested, the following microbial groups, usually considered indicators of contamination, were estimated. Sulfite-reducing clostridia (*Clostridium perfringens*), using SPS (Sulfite Polymyxin Sulfadiazine) agar as the medium, were incubated at 37 °C for 48 h. The potential presence of the *Salmonella* spp. pathogen was investigated according to the following protocol: pre-enrichment stage (incubation of the sample at 37 °C for 18 h in buffered peptone water, where the meal-to-nutrient medium ratio was 1:9); enrichment stage (incubation in Rappaport–Vassiliadis broth, a selective medium, at 41.5 °C for 24 h, extendable to 48 h in the case of initial negative results); selective isolation stage (seeding from the presumptive positive selection medium grown on Hektoen Agar incubated at 37 °C for 24 h); and confirmation stage (study of suspected colonies by growth characteristics on Kligler Agar, incubated at 37 °C for 24 h). The presence of Enterobacteria was investigated in VRBG agar plates grown for 24 h at 37 °C. The presence of total aerobic mesophilic bacteria (TAMB) was investigated in PCA agar plates grown for 24 h at 37 °C.

### 2.7. Statistical Analyses

The experimental results were expressed as mean ± SD. Statistical differences in the insect digestibility between the slaughtering and drying temperature were analyzed with the ANOVA multivariate test, followed by a comparison of means (Tukey test). The proximate composition, OMd, DH/NH_2_, DH/100 g DM, TH, and hygienic–sanitary results were analyzed using simple ANOVA followed by a multiple comparison of means (Tukey test). The correlations were analyzed using multivariate pairwise correlation analysis. A normality test was performed. The data did not follow a normal distribution; so, data normalization was performed using a two-step transformation (first, the variable was transformed into a percentile rank and followed by the inverse-normal transformation) [33]. The normal distribution of the residuals and the transformed data was confirmed. The tests were carried out using IBM SPSS Statistics software (29.0.1.0). For graphic representation, all the data in tables and figures were kept in raw form (prior to normalization) for clarity of presentation.

## 3. Results

### 3.1. Proximate Composition

Regarding the proximate composition (Table 2 and Table 3), no significant differences were found in the protein data from the different slaughtering and drying temperatures of BSFL. The slaughter methods did not show significant differences in ADF (Table 2). On the other hand, ADF was affected by drying temperature, showing the highest value at 50 and the lowest at 70 °C. The ash content was higher for the M treatment, while no significant differences were found with the other slaughter methods. Drying at 50 °C showed the highest ash data.

### 3.2. Organic Matter Digestibility (OMd)

The OMd results, according to multivariate statistics (Table 4 and Table 5), showed that there were significant differences in terms of the slaughter method, and the frozen method had the lowest data. Significant differences were also found in terms of the drying temperature, with the highest results obtained at 70 °C.

**Figure 1:**
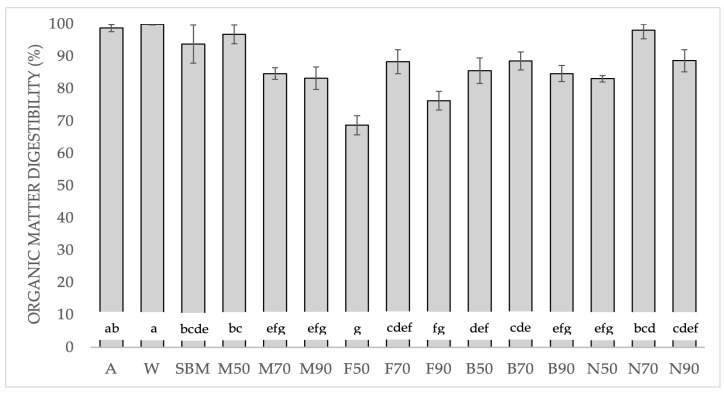
Organic matter digestibility (%) of the BSFL pretreatments, soybean meal, albumin, and whey protein, and the relative standard deviation (±SD). Significant differences in the Tukey test are represented by different letters (*p* < 0.05). A: albumin; W: whey protein; SBM: soybean meal; M: Melacide^®^; F: frozen; B: blanched; N: nitrogen; 50, 70, 90: dried at 50 °C, 70 °C, 90 °C.

When the combined effect was analyzed, the simple ANOVA (Figure 1) showed high digestibility for M50 and N70, which achieved results similar to the albumin and whey protein.

### 3.3. Hydrolysis Degree (DH/NH_2_) and Total Hydrolysis (TH)

Regarding the slaughter methods, there were differences between DH/NH_2_ and TH (Table 6 and Table 7). The blanched and nitrogen methods showed the highest DH/NH_2_; however, the nitrogen method showed the lowest TH. Therefore, if both parameters were considered, the blanched method would be the slaughter method with the best results. Furthermore, when analyzing the effect of drying temperature, the highest values for DH/NH_2_ were obtained at 70 °C. However, TH was not affected by temperature (Table 6).

**Figure 2:**
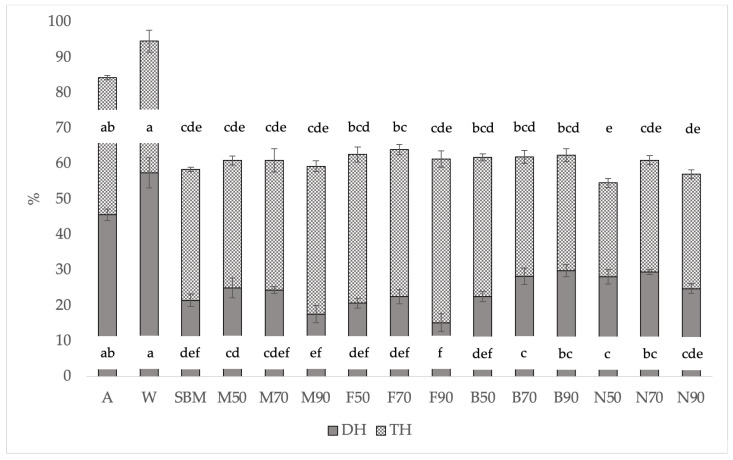
DH (% of total amino groups) and total hydrolysis (% of total amino groups) of the BSFL pretreatments, fishmeal, soybean meal, albumin, and whey protein, and the relative standard deviation (±SD). Significant differences in the Tukey test are represented by different letters (*p* < 0.05). A: albumin; W: whey protein; SBM: soybean meal; M: Melacide^®^; F: frozen; B: blanched; N: nitrogen; 50, 70, 90: dried at 50 °C, 70 °C, 90 °C; DH: hydrolysis degree; TH: total hydrolysis.

When analyzing the combination of treatments (Figure 2), the highest DH values were obtained for B90 and N70, which outperformed SBM, while the worst were M90 and F90. However, when analyzing TH, these differences were clearly attenuated, with the results among treatments being equal, and only N50 showed lower hydrolysis.

### 3.4. Gastric and Intestinal Digestion

The amino groups were observed prior to the onset of hydrolysis (Table 8 and Table 9).

Considering the drying temperature data at the end of the hydrolysis, the lowest results were obtained at 90 °C. Regarding the method of slaughter, the highest values of hydrolysis during gastric digestion were obtained for the blanching method, while in intestinal digestion, the highest values of hydrolysis corresponded to the samples treated with nitrogen.

When taking into account the combination of treatments, the samples matched the SBM results at the end of the hydrolysis, with the exception of B50, M (F50, F70, F90), and F (F50, F70, F90), which showed a significantly lower amount of free amino groups.

### 3.5. Correlations

As shown in Table 10, the OMd was positively correlated with the DH and TH. Furthermore, there was also a positive correlation between CP and the different protein digestibility measurements. Additionally, the protein digestibility variables were positively correlated (r = 0.456).

Furthermore, we obtained a negative correlation between ADF-DH (r = −0.489) and ADF-TH (r = −0.385).

### 3.6. Hygienic–Sanitary Quality Study: Microbial Contamination Indicators

The results obtained (Table 11) showed a clear influence of the treatments applied on the levels of microbial groups indicative of contamination present in insects. The clearest case was that of the Enterobacteriaceae group, as all the combinations tested led to the disappearance of this type of bacteria, regardless of the method of slaughter or the drying temperature. The latter generated the lowest degree of Thermophilic Acidophilic Bacteria (TAMB) reduction because of temperature action. The presence of *Salmonella* and enterobacteria was also safely controlled by the drying temperature, regardless of the slaughter method applied. However, in this case, the thermal value required was somewhat higher, as treatments performed at 50 °C were not sufficient to promote the disappearance of this pathogen. Finally, and in relation to SRCs, the only problematic batch was that of the insects slaughtered with liquid nitrogen.

## 4. Discussion

To realize the full potential of insects as novel and sustainable food in the near future, the insect mass-rearing industry needs to develop and optimize insect meal processing systems to maximize their nutritional potential and food safety. The high variability observed in the nutritional composition of the same insect species shows that, in addition to other factors, the slaughter and drying method can clearly affect nutritional composition. All this makes it necessary to investigate the effects of common processing, slaughtering, and drying conditions on the nutritional characteristics and digestive proteolysis of BSLF meal.

It is clear that the slaughter method and the drying temperature do not affect the protein content of BSLF (Table 3). However, drying at 70 °C showed a lower proportion of fiber and ash, and as pointed out by several studies [34,35], higher levels of fiber could be indicative of worse digestibility.

OMd has traditionally been used to assess the digestibility of food [36]. Based on our OMd results (Figure 1), the frozen method was the worst, and 70 °C was the best drying temperature. In general, when combining both processes, our data were similar to those obtained by Bosch et al. [37], who obtained 84.3% for BSFL and poultry meat meal (85.8%) despite being freeze-dried insect samples. However, except for N70 (liquid nitrogen-dried at 70 °C) (98.1%), they were less than soybean meal (93.8%).

The DH data (Table 7) showed higher results for the nitrogen and blanched slaughter methods. The frozen and nitrogen methods are both freezing slaughter treatments; however, they differ in the freezing temperature and, therefore, the speed of slaughter, as nitrogen slaughter is instantaneous. This could explain the differences in their results. As it is a fast slaughter, insects do not have time to develop stress response strategies, such as enzymatic browning, which can hinder protein hydrolysis. Browning can affect protein digestibility and quality and seems to be related to protein aggregation due to insect stress [20]. According to Janssen et al. [29], five enzymes may play an important role in enzymatic browning in insects as follows: phenol oxidase, laccase, tyrosine hydroxylase, DOPA decarboxylase, and peroxidase. These are related to defense mechanisms and react quickly to stress. They are also involved in the sclerotization of the exoskeleton. Wessels et al. [38] noted that, of these, phenol oxidase is the key to browning and is likely to remain active after freezing. This is because there are anti-freeze proteins in insects that ensure that part of the water does not finish freezing, and enzymatic reactions that favor the formation of aggregates and browning continue to take place. This could explain the higher DH data in the nitrogen method with respect to the frozen method, as well as the higher results for the blanched method (Table 7). Some studies confirm the improvement in digestibility using blanching, such as that by Leni et al. [20], who obtained higher DH results for BSFL in blanched (32%) than in frozen (16.5%) insects. In the industry, blanching has been used to inactivate enzymes and preserve food quality. This is because blanching blocks enzymatic browning by inactivating phenol oxidase [20]. Different studies have shown less browning with blanching than with traditional freezing slaughter (−18 °C) [20,30,39]. Other studies found no improvement in digestibility with blanching [27,40,41]. Even in Janssen et al. [19], the DH data for *Alphitobius diaperinus* were the same in the raw and blanched insects but did not improve the digestibility in BSFL or *Tenebrio molitor.*

As indicated in the Materials and Methods Section, in this experiment, we used Melacide^®^ to try to reduce the possible enzymatic browning of the insects. To date, no studies using Melacide^®^ in insects have been found in the literature. The DH data showed higher results with Melacide^®^ (M) than without Melacide^®^ (F) (Table 7); therefore, it is a desirable treatment if slaughter is to be carried out by freezing.

Regarding drying temperatures, the highest DH was obtained at 70 °C and the lowest when increasing the temperature to 90 °C (Table 7). These data support those obtained by Mancini et al. [40], who obtained a better digestibility for TM in a traditional oven at 70 °C than at 150 °C. High heat treatments appear to make proteins more compact by polymerizing them [18]. Janssen et al. [19] observed that this denaturation and aggregation reduced the accessibility of digestive enzymes, decreasing protein digestibility.

When the treatments were compared with soybean meal, all showed similar or even higher DH including the blanched samples dried at 70 and 90 °C (B70 and B90) and the liquid nitrogen samples dried at 50 °C and 90 °C (N50 and N70) (Figure 2).

However, in the TH data, which considers DH plus other small peptides that have been solubilized after hydrolysis and are expected to be digestible, the statistical differences disappeared, showing similar digestibility among almost all treatments. TH was higher in the treatments with lower DH (Figure 2). This could indicate a slower velocity of digestion for these samples, maybe because they have a higher content and/or a less digestible structure of the scleroprotein in their exoskeleton. However, the TH data indicate that these differences are due to small peptides not accounted for by DH, which are likely to be absorbed. This may be relevant if we want to value these insect meals as feed. Protein digestibility may be underestimated if only DH is taken into account. Differences were found between slaughter with nitrogen and the rest of the treatments. Regarding the drying temperature, again, higher results were obtained at 70 °C (Table 7).

When evaluating the free amino groups per g of sample (in DM), high initial values were observed (Table 9). This may indicate that the endogenous enzymes of the insect are active from the beginning. Therefore, as these enzymes are inactivated by blanching (B), this method of slaughter releases the fewest amino groups. Leni et al. [42] observed partial solubilization of insect protein under hydrolysis conditions without enzymatic action below 50%. As stated above, these data were higher than those obtained by Hall et al. [43] (20% for cricket) and similar to those of a previous study by Caligiani et al. [8] (43% for BSFL). They suggested that these differences may depend on the temperature, time, pH, and enzyme conditions. After the gastric and intestinal phases, it seems clear that the slaughter method with the lowest release of amino groups was the frozen method, and the methods with the highest release were the blanched and nitrogen (Table 9).

The decrease in amino group content with increasing drying temperature (Table 9) could be due to an aggregation of proteins that hinders their breakage and, therefore, the release of their amino groups. It is known that increasing or decreasing temperature destabilizes proteins and can promote their aggregation [44]. Temperatures above 60–70 °C can destroy hydrogen bonds and electrostatic and hydrophobic interactions among proteins and disorganize their structure, thus favoring protein denaturation and aggregation [45]. Santiago et al. [46] studied protein aggregation in *Gryllus assimilis* and observed that aggregation progressively increased from practically non-existent at 65 °C to its maximum at 90–95 °C. Janssen et al. [19] linked protein denaturation and aggregation with worsening digestibility, as aggregates hinder the action of enzymes for protein degradation.

After the gastric and intestinal phases, there was a higher percentage of amino groups released in albumin and whey protein as expected. In general, the BSFL meals had similar protein yields for the same amount of dry matter as soybean meal, and only a few BSFL treatments, including Melacide^®^ dried at 90 °C (M90), frozen (F50, F70, F90), and blanched-dried at 50 °C (B50), showed significantly lower data (Table 9).

The diversity in the way authors approach protein digestibility makes it difficult to compare results. Therefore, we consider it of interest to study the potential correlation between the different parameters. A positive correlation was found between the CP and different protein digestibility measurements (Table 10). This could indicate that the higher the protein content, the higher the protein digestibility. According to Finke [34], the ADF of insects consists of chitin and associated cuticular proteins, and since the determination of chitin is complex, ADF can be used to estimate digestibility. Protein digestibility is affected because chitin is neither degraded nor absorbed in the digestive tract [47]. Similar to us, Marono et al. [35] found a negative correlation between ADF and digestibility for BSFL. Therefore, although it is not a parameter that measures digestibility, when detailed studies are not possible, determining ADF can be a rough predictive tool for the degree of protein digestibility of the insect. In general, ash and ADF showed a negative correlation with the different protein digestibility parameters analyzed; nevertheless, further research is required to confirm this result. The correlation between CP and TH or DH showed that both parameters can be used interchangeably.

The presence of certain microbial species in the final products made from insects and destined for the food market is of great importance from a hygienic–sanitary perspective. In this sense, guaranteeing the suitability of the processing protocols applied to ensure their microbial quality gives these protocols additional value in the food sector. The efficiency in the progressive disappearance of the Enterobacteriaceae group seems to be mainly a consequence of the application of drying temperatures, as noted in other studies [48], which describe the high efficiency of thermal protocols, even with low potential, to control the presence of this type of bacteria. In the case of TAMB, the observed impact was not as considerable, although the reductions in the counts obtained were more significant as the drying temperature increased (Table 11). Thus, the important role of temperature in relation to the microbiological quality of the insect seems to be confirmed. However, the method of slaughter also contributed to the observed differences, with nitrogen treatment being the most favorable compared with freezing. Treatments performed at 50 °C were not sufficient to ensure the avoidance of *Salmonella* (Table 11). Therefore, it is important to ensure the correct application of the treatment; otherwise, the resistance of this bacterium tends to increase [49]. Although there are few studies that combine the study of microbial quality with slaughter techniques based on the use of liquid nitrogen, the presence of SRC in insects processed by this type of methodology has been described [50]. Taking into account what was mentioned, and from a microbial perspective, it seems logical to conclude that the temperature applied during the drying stage is more decisive than the method of slaughtering. Temperatures of approximately 70 °C should ensure the hygienic–sanitary quality of insects in terms of their use in the food sector.

## 5. Conclusions

In this study, ADF and DH/100 g DM were correlated with DH/NH_2_ and TH. As these parameters are faster and simpler, they could be useful in cases where a very detailed study on digestibility is not necessary or where there are time or economic limitations.

Regarding slaughter methods, blanching and liquid nitrogen showed higher results than freezing and Melacide^®^, both in terms of protein digestibility and hygienic quality. However, in terms of economy and easy accessibility, blanching is more advisable.

The drying temperature plays an important role. Generally, 70 °C resulted in better digestibility data than 50 °C. At 90 °C, the lowest results were obtained because, at higher temperatures, there was a risk of protein aggregation processes that would hinder digestibility.

Therefore, this study shows that the protein digestibility results of black soldier fly larvae are higher when slaughtering by blanching or freezing with liquid nitrogen and drying at 70 °C. Moreover, this temperature is sufficient to maintain a good hygienic–sanitary quality of the black soldier fly meals.

## Figures and Tables

**Table 1:** Treatments according to the slaughter method and drying temperature.

Slaughtering	Frozen			Blanched + Frozen			Melacide^®^ + Frozen			Liquid Nitrogen		
T dried (°C)	50	70	90	50	70	90	50	70	90	50	70	90
Treatment	F50	F70	F90	B50	B70	B90	M50	M70	M90	N50	N70	N90

**Table 2:** MANOVA and ANOVA fit statistics for CP, ADF, and ash in the BSFL treatments, soybean meal, albumin, and whey protein. CP: crude protein; ADF: acid detergent fiber.

	Factor	F	df	*p*-Value	Test
CP	Temperature	0.029	2	0.971	Multivariate ANOVA
	Sacrifice	0.234	3	0.872	
	T × S	0.767	6	0.604	
ADF	Temperature	32.662	2	<0.001	
	Sacrifice	0.717	3	0.552	
	T × S	6.952	6	<0.001	
Ash	Temperature	48.620	2	<0.001	
	Sacrifice	28.390	3	<0.001	
	T × S	3.905	6	<0.001	
CP	-	4.772	14	<0.001	Simple ANOVA
ADF	-	25.780	14	<0.001	
Ash	-	31.305	14	<0.001	

**Table 3:** Proximate composition (g/100 g dry matter) of the BSFL treatments, soybean meal, albumin, and whey protein, and the relative standard deviation (±SD). Significant differences in the Tukey test (*p* < 0.05) are represented by different letters. A: albumin; W: whey protein; SBM: soybean meal; M: Melacide^®^; F: frozen; B: blanched; N: nitrogen; 50, 70, 90: dried at 50 °C, 70 °C, 90 °C; CP: crude protein; ADF: acid detergent fiber.

		CP	ADF	Ash	Test
Slaughtering	Melacide^®^	nsd	nsd	16.2 ± 1.8 ^a^	Multivariate ANOVA
	Frozen	nsd	nsd	13.8 ± 1.7 ^b^	
	Blanched	nsd	nsd	14.0 ± 1.6 ^b^	
	Nitrogen	nsd	nsd	13.6 ± 0.3 ^b^	
Temperature	50 °C	nsd	5.8 ± 0.8 ^a^	16.0 ± 1.9 ^a^	
	70 °C	nsd	4.6 ± 0.3 ^c^	13.6 ± 1.3 ^b^	
	90 °C	nsd	5.1 ± 0.5 ^b^	13.6 ± 0.7 ^b^	
Sample	A	98.9 ± 0.9 ^a^	0.0 ± 0.0 ^g^	1.1 ± 0.0 ^h^	Simple ANOVA
	W	90.5 ± 1.3 ^b^	0.0 ± 0.0 ^g^	3.2 ± 0.1 ^gh^	
	SBM	50.0 ± 0.5 ^c^	8.4 ± 0.8 ^a^	6.9 ± 0.0 ^fgh^	
	M50	37.2 ± 3.5 ^d^	6.1 ± 0.3 ^bc^	18.6 ± 0.3 ^a^	
	M70	34.7 ± 3.3 ^d^	4.3 ± 0.2 ^fg^	15.5 ± 0.8 ^bc^	
	M90	34.0 ± 0.5 ^d^	5.2 ± 0.5 ^def^	14.7 ± 0.0 ^bcd^	
	F50	34.9 ± 1.7 ^d^	6.6 ± 0.3 ^b^	15.7 ± 1.5 ^b^	
	F70	34.6 ± 1.3 ^d^	4.5 ± 0.3 ^fg^	12.6 ± 0.8 ^efgh^	
	F90	33.9 ± 0.4 ^d^	4.9 ± 0.5 ^def^	13.2 ± 0.3 ^defg^	
	B50	34.2 ± 1.5 ^d^	5.9 ± 0.3 ^bcd^	16.0 ± 0.2 ^b^	
	B70	34.6 ± 0.4 ^d^	4.9 ± 0.5 ^def^	12.7 ± 0.6 ^efgh^	
	B90	35.8 ± 1.8 ^d^	4.9 ± 0.4 ^def^	13.3 ± 0.2 ^defg^	
	N50	34.8 ± 1.3 ^d^	4.6 ± 0.1 ^efg^	13.8 ± 0.1 ^bcde^	
	N70	34.5 ± 0.2 ^d^	4.7 ± 0.1 ^ef^	13.5 ± 0.5 ^cdef^	
	N90	34.8 ± 0.6 ^d^	5.5 ± 0.4 ^cde^	13.3 ± 0.0 ^defg^	

**Table 4:** MANOVA fit statistics of the organic matter digestibility in the BSFL treatments. ANOVA fit statistics of the organic matter digestibility in the BSFL treatments, albumin, whey protein, and soybean meal. OMd: organic matter digestibility.

	Factor	F	df	*p*-Value	Test
OMd	Temperature	10.635	2	<0.001	Multivariate ANOVA
	Sacrifice	10.705	3	<0.001	
	T × S	10.423	6	<0.001	
	-	19.395	14	<0.001	Simple ANOVA

**Table 5:** Organic matter digestibility of the BSFL treatments and the relative standard deviation (±SD). Significant differences in the Tukey test (*p* < 0.05) are represented by different letters. OMd: organic matter digestibility.

		OMd	Test
Slaughtering	Melacide^®^	88.3 ± 6.9 ^a^	Multivariate ANOVA
	Frozen	77.8 ± 9.0 ^b^	
	Blanched	86.3 ± 3.2 ^a^	
	Nitrogen	90.0 ± 6.9 ^a^	
Temperature	50 °C	83.6 ± 10.7 ^b^	
	70 °C	89.9 ± 5.7 ^a^	
	90 °C	83.3 ± 5.4 ^b^	

**Table 6:** MANOVA fit statistics for the hydrolysis degree and total hydrolysis in the BSFL treatments. ANOVA fit statistics for the hydrolysis degree and total hydrolysis in the BSFL treatments, albumin, whey protein, and soybean meal.

	Factor	F	df	*p*-Value	Test
DH	Temperature	11.781	2	<0.001	Multivariate ANOVA
	Sacrifice	32.258	3	<0.001	
	T × S	8.391	6	<0.001	
TH	Temperature	3.273	2	0.057	
	Sacrifice	6.224	3	0.003	
	T × S	1.341	6	0.281	
DH	-	26.423	14	<0.001	Simple ANOVA
TH	-	8.875	14	<0.001	

**Table 7:** Hydrolysis degree and total hydrolysis among different slaughtering methods and drying temperatures. Significant differences in the Tukey test (*p* < 0.05) are represented by different letters. DH: hydrolysis degree; TH: total hydrolysis.

		DH	TH	Test
Slaughtering	Melacide^®^	22.4 ± 3.9 ^b^	60.4 ± 2.3 ^ab^	Multivariate ANOVA
	Frozen	19.5 ± 3.7 ^c^	62.6 ± 1.9 ^a^	
	Blanched	26.9 ± 3.8 ^a^	62.0 ± 1.5 ^a^	
	Nitrogen	27.5 ± 2.8 ^a^	57.6 ± 3.2 ^b^	
Temperature	50 °C	24.1 ± 3.3 ^b^	nsd	
	70 °C	26.2 ± 3.6 ^a^	nsd	
	90 °C	21.9 ± 6.2 ^b^	nsd	

**Table 8:** MANOVA fit statistics of the amino groups (g/100 g DM) in the BSFL treatments. ANOVA fit statistics of the amino groups (g/100 g DM) in the BSFL treatments, albumin, whey protein, and soybean meal.

	Factor	F	df	*p*-Value	Test
Initial (0′)	Temperature	47.222	2	<0.001	Multivariate ANOVA
	Sacrifice	15.712	3	<0.001	
	T × S	4.721	6	0.003	
Gastric (240′)	Temperature	0.300	2	0.744	
	Sacrifice	23.388	3	<0.001	
	T × S	7.631	6	<0.001	
Intestinal (480′)	Temperature	12.982	2	<0.001	
	Sacrifice	42.667	3	<0.001	
	T × S	8.487	6	<0.001	
Initial (0′)	-	19.021	14	<0.001	Simple ANOVA
Gastric (240′)	-	24.055	14	<0.001	
Intestinal (480′)	-	26.855	14	<0.001	

**Table 9:** Amino groups (g/100 g DM) of the BSFL treatments, soybean meal, albumin, and whey protein at the start and end of the gastric and intestinal phases of protein hydrolysis and the relative standard deviation (±SD). Significant differences in the Tukey test (*p* < 0.05) obtained using the different ANOVAs are represented by different letters. A: albumin; W: whey protein; SBM: soybean meal; M: Melacide^®^; F: frozen; B: blanched; N: nitrogen; 50, 70, 90: dried at 50 °C, 70 °C, 90 °C; nsd: no significant differences.

		Initial (0′)	Gastric (240′)	Intestinal (480′)	Test
Slaughtering	Melacide^®^	4.5 ± 0.9 ^a^	6.1 ± 2.0 ^b^	8.4 ± 1.5 ^c^	Multivariate ANOVA
	Frozen	4.2 ± 0.4 ^b^	4.6 ± 0.3 ^c^	7.2 ± 1.4 ^d^	
	Blanched	3.3 ± 0.9 ^c^	8.3 ± 1.1 ^a^	9.8 ± 1.3 ^b^	
	Nitrogen	4.2 ± 0.9 ^b^	6.5 ± 1.0 ^b^	11.2 ± 1.1 ^a^	
Temperature	50 °C	4.9 ± 0.4 ^a^	nsd	9.2 ± 1.6 ^ab^	
	70 °C	4.1 ± 0.8 ^b^	nsd	9.9 ± 1.6 ^a^	
	90 °C	3.2 ± 0.5 ^c^	nsd	8.3 ± 2.4 ^b^	
Sample	A	4.0 ± 0.1 ^cdef^	18.8 ± 0.5 ^ab^	39.3 ± 0.9 ^ab^	Simple ANOVA
	W	4.7 ± 0.1 ^abc^	18.5 ± 0.8 ^a^	46.0 ± 1.5 ^a^	
	SBM	1.8 ± 0.0 ^g^	9.4 ± 0.2 ^abc^	11.2 ± 1.6 ^cd^	
	M50	5.1 ± 0.1 ^ab^	8.6 ± 0.7 ^cde^	9.4 ± 0.5 ^def^	
	M70	5.1 ± 0.4 ^a^	4.6 ± 0.3 ^g^	9.3 ± 1.0 ^def^	
	M90	3.4 ± 0.1 ^defg^	5.0 ± 0.6 ^fg^	6.6 ± 0.6 ^fg^	
	F50	4.7 ± 0.1 ^abc^	4.5 ± 0.0 ^g^	7.6 ± 0.7 ^efg^	
	F70	4.2 ± 0.2 ^cde^	4.6 ± 0.4 ^g^	8.3 ± 1.0 ^efg^	
	F90	3.7 ± 0.1 ^cdefg^	4.7 ± 0.4 ^fg^	5.6 ± 0.5 ^g^	
	B50	4.4 ± 0.4 ^bcd^	6.8 ± 0.2 ^defg^	8.2 ± 0.5 ^efg^	
	B70	3.1 ± 0.2 ^efg^	8.9 ± 0.2 ^cde^	10.2 ± 0.8 ^cde^	
	B90	2.4 ± 0.1 ^fg^	9.1 ± 0.1 ^bcd^	10.8 ± 0.8 ^cd^	
	N50	5.3 ± 0.3 ^a^	6.4 ± 0.4 ^defg^	11.5 ± 0.6 ^bcd^	
	N70	3.9 ± 0.2 ^cdef^	7.3 ± 0.3 ^cdef^	12.0 ± 0.9 ^bc^	
	N90	3.3 ± 0.3 ^defg^	5.6 ± 1.2 ^efg^	10.0 ± 0.9 ^cde^	

**Table 10:** Correlations among proximal composition, organic matter digestibility, and protein digestibility (DH and TH). The correlation is significant at the 0.05 level. CP: crude protein; ADF: acid detergent fiber; OMd: organic matter digestibility; DH: hydrolysis degree; TH: total hydrolysis; p: *p*-value.

	TH	p	DH	p	OMd	p	Ash	p	ADF	CP
CP	0.329	0.031	0.479	0.001	0.607	<0.001	−0.408	0.006	nsd	-
ADF	−0.385	0.011	−0.489	<0.001	−0.383	0.011	0.450	0.002	-	-
Ash	−0.302	0.047	−0.374	0.012	nsd	-	-	-	-	-
OMd	0.454	0.002	0.636	<0.001	-	-	-	-	-	-
DH	0.456	0.002	-	-	-	-	-	-	-	-

**Table 11:** Hygienic–sanitary quality, according to the levels of microbial contamination indicators, of insects after processing according to the different treatments. Values are expressed as Log_10_ CFU mL^−1^. Mean values ± SDs are shown. Significant differences in the Tukey test are represented by different letters (*p* < 0.05). M: Melacide^®^; F: frozen; B: blanched; N: nitrogen; 50, 70, 90: dried at 50 °C, 70 °C, 90 °C. TAMB: thermophilic acidophilic bacteria; SRC: sulfite-reducing clostridia. * Detection limit: 1 log CFU mL^−1^.

	TAMB	Enterobacteria	SRC *	*Salmonella*
Control	5.92 ± 0.35 ^ab^	5.32 ± 0.13 ^a^	Negative	Positive
M50	7.25 ± 0.33 ^a^	<1 ^b^	Negative	Positive
M70	2.92 ± 2.53 ^cde^	<1 ^b^	Negative	Negative
M90	1.65 ± 1.43 ^ef^	<1 ^b^	Negative	Negative
F50	6.14 ± 0.25 ^ab^	<1 ^b^	Negative	Positive
F70	4.84 ± 0.50 ^bc^	<1 ^b^	Negative	Negative
F90	2.11 ± 1.92 ^de^	<1 ^b^	Negative	Negative
B50	6.97 ± 0.13 ^a^	<1 ^b^	Negative	Positive
B70	3.54 ± 0.92 ^cd^	<1 ^b^	Negative	Negative
B90	<1 ^f^	<1 ^b^	Negative	Negative
N50	1.71 ± 0.06 ^ef^	<1 ^b^	Positive	Negative
N70	<1 ^f^	<1 ^b^	Positive	Negative
N90	<1 ^f^	<1 ^b^	Positive	Negative

## Data Availability

The data are available in a publicly accessible repository. The data presented in this study are openly available in Repositorio Institucional de la Universidad de Almería at http://hdl.handle.net/10835/14827, accessed on 29 April 2024.
